# Left ventricular pseudo-false aneurysm after ventricular septal dissection closure: a case report

**DOI:** 10.1186/s44215-024-00139-5

**Published:** 2024-02-27

**Authors:** Ryo Yamaguchi, Satoshi Ohki, Kiyomitsu Yasuhara, Shuichi Okonogi, Ayako Nagasawa, Takao Miki, Yusuke Kato, Tamiyuki Obayashi

**Affiliations:** Department of Cardiovascular Surgery, Isesaki Municipal Hospital, Isesaki, Gunma 372-0817 Japan

**Keywords:** Pseudo-false aneurysm, Ventricular septal dissection, Myocardial infarction

## Abstract

**Background:**

Left ventricular pseudo-false aneurysm is a rare complication of myocardial infarction and generally caused by an intramyocardial dissecting hematoma due to a fragile myocardium. The serpiginous dissecting case of ventricular septal perforation has an entry port in the left ventricle and exit port in the right ventricle, and the entry port must be closed to leave the dissected chamber on the low-pressure right side for treatment. Herein, we report a case of a large left ventricular pseudo-false aneurysm that was unaccompanied by a shunt after the surgical repair of a ventricular septal dissection.

**Case presentation:**

A 72-year-old woman underwent percutaneous coronary intervention to the right coronary artery; 3 days later, she was urgently referred to our hospital with ventricular septal perforation. The patient was treated with sandwich patch repair via a right ventricular incision. Postoperative transthoracic echocardiography revealed no residual shunt. However, 3 months postoperatively, enhanced chest computed tomography revealed a large left ventricular pseudo-false aneurysm bulging on the right ventricular side, causing congestive heart failure. An intra-aortic balloon pump was inserted for treatment. In our case, the left ventricular pseudo-false aneurysm was caused by the closure of only the exit port in the right ventricle and insufficient closure of the entry port in the left ventricle during ventricular septal dissection. Therefore, we closed the entry port through a pseudo-false aneurysm using a Dacron patch during the second surgery.

**Conclusions:**

Recognizing and identifying the ventricular septal dissection after myocardial infarction are crucial for providing the best treatment and surgical approaches. When ventricular septal dissection is treated using sandwich patch repair via a right ventricular incision, the entry port in the left ventricle must be securely closed with a large patch using transmural mattress sutures.

## Background

Ventricular septal perforation (VSP) complicates 1–2% of all patients with myocardial infarction, and most cases require a prompt surgical approach [1]. Ventricular septal dissection is a less common complication. Serpiginous dissection and rupture cases have an entry port in the left ventricle (LV) and exit port in the right ventricle (RV); moreover, surgery is recommended to close the entry port, leaving the chamber on the low-pressure right side for treatment [2]. We report a case of a large left ventricular pseudo-false aneurysm that was unaccompanied by a shunt after the surgical repair of a ventricular septal dissection. The aneurysm was caused by insufficient closure of the entry port in the LV during the ventricular septal dissection. Therefore, we closed the entry port through a pseudo-false aneurysm using a Dacron patch during the second surgery. Recognizing and identifying the ventricular septal dissection after myocardial infarction are crucial for providing the best treatment and surgical approaches. When ventricular septal dissection is treated using sandwich patch repair via a right ventricular incision, the entry port in the LV must be securely closed with a large patch using transmural mattress sutures. This case highlights the importance of surgical planning and techniques for ventricular septal dissection. Informed consent was obtained from the patient for the publication of this case report.

## Case presentation

**Fig. 1:**
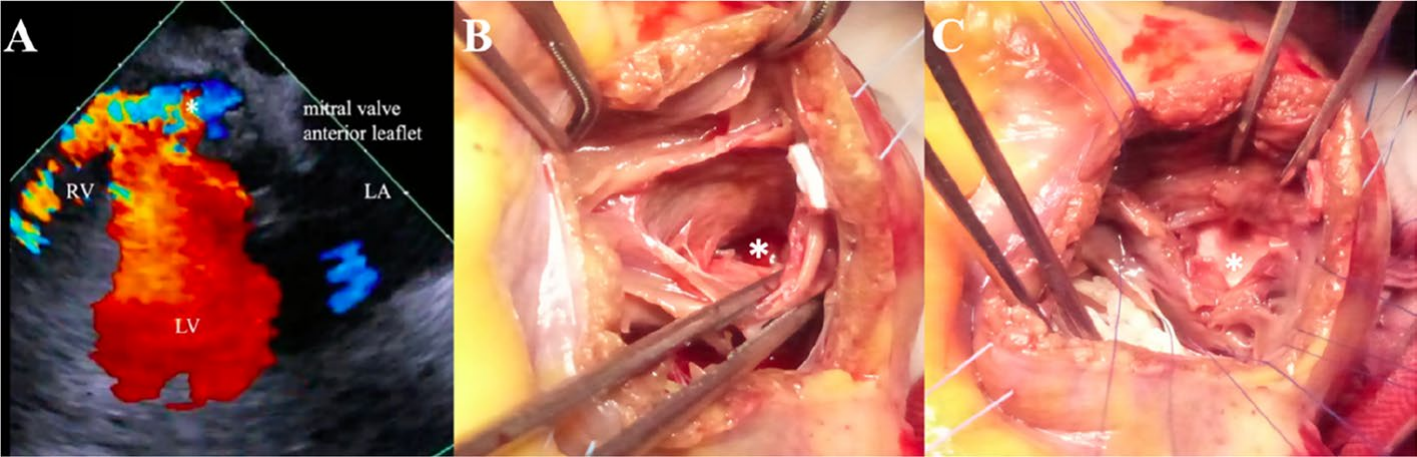
**A** Transesophageal echocardiography revealed a ventricular septal dissection near the mitral valve annulus (star) and a left-to-right shunt. **B** When the right ventricular incision, a ventricular septal perforation in the posterior wall was confirmed (star). **C** The first Dacron patch was inserted through a ventricular septal perforation

A 72-year-old woman was referred to our hospital for ventricular septal perforation 3 days after successful percutaneous coronary intervention of the right coronary artery with a drug-eluting stent. Preoperative transesophageal echocardiography revealed serpiginous dissection with an entry port around the mitral valve in the LV and exit port in the RV (Fig. 1A). The patient underwent sandwich patch repair via a right ventricular incision because of postinfarction interventricular septal dissection and perforation (Fig. 1B and C). Postoperative transthoracic echocardiography revealed no residual shunt, and the patient was discharged day 21 postoperatively.

**Fig. 2:**
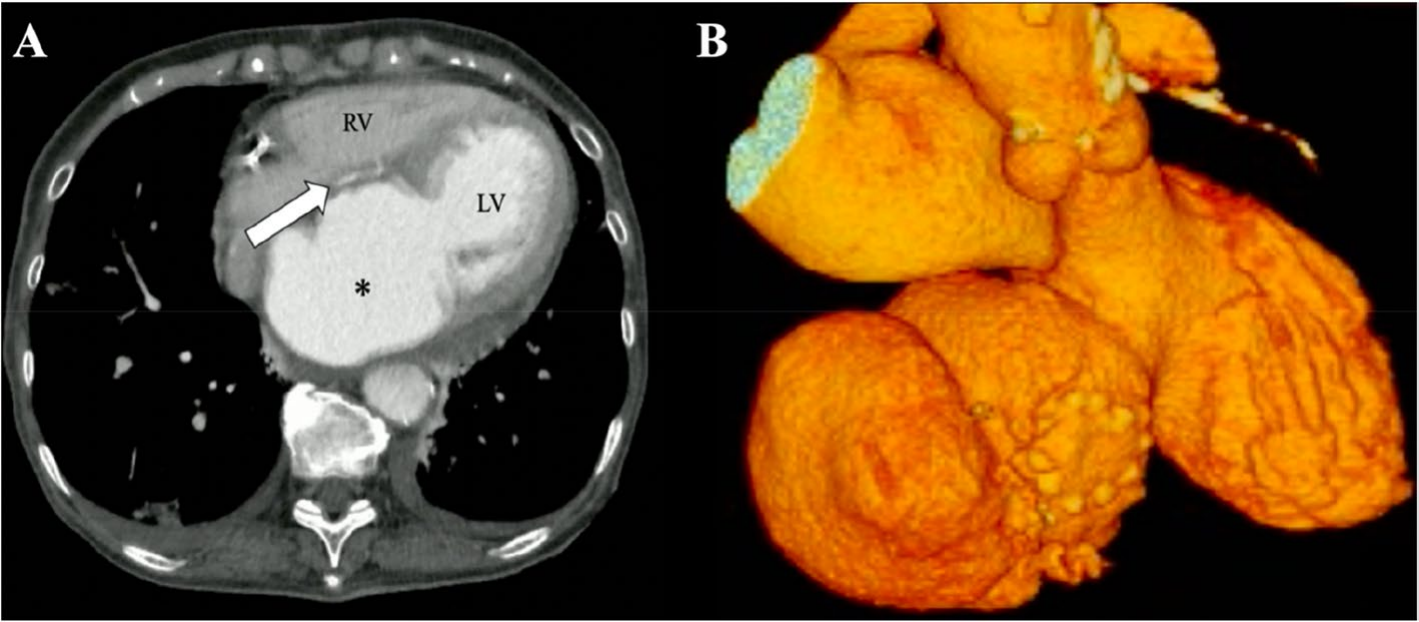
Axial (**A**) and 3-dimensional (**B**) enhanced computed tomography showed a large left ventricular septal aneurysm (star), and a patch of the right ventricle in the first operation could be seen on the anterior surface of the ventricular septal aneurysm (arrow)

At 3 months postoperatively, the patient was admitted with rapidly deteriorating severe breathlessness in a preshock state and acute heart failure. Electrocardiography revealed abnormal Q waves in leads II, III, and aVF. However, laboratory findings demonstrated that creatine kinase (CK) and CK-MB levels were within the normal range. Transthoracic echocardiography revealed no residual shunt; however, aneurysmal enlargement of the septal wall was present. Circulatory support with an intra-aortic balloon pumping (IABP) catheter was initiated because of unstable hemodynamics. Enhanced computed tomography (CT) revealed a large left ventricular septal aneurysm bulging on the RV (Fig. 2A and B). We believe that the left ventricular pseudo-false aneurysm was caused by insufficient closure of the entry port in the LV during the first surgery.

**Fig. 3:**
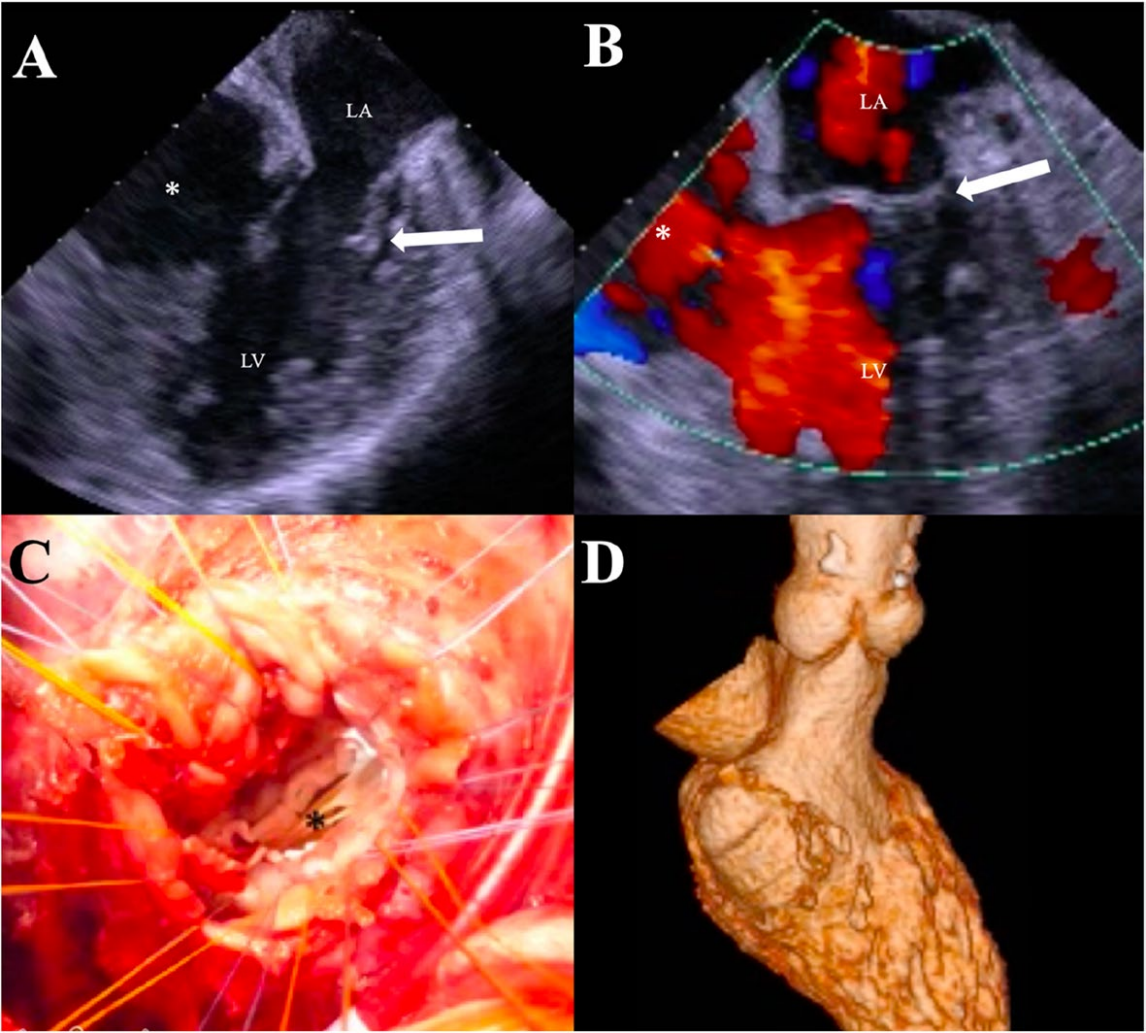
Transesophageal echocardiography revealed the left ventricular septal aneurysm (star) was near the mitral valve (arrow). **A** was diastole, and **B** was systole. **C** When the left ventricular septal aneurysm was incised, the entry port and left ventricular papillary muscle were confirmed (star). **D** Postoperative-enhanced CT revealed no pseudo-false aneurysm between left and right ventricle

Subsequently, an emergency surgery was performed. Transesophageal echocardiography revealed the left ventricular septal aneurysm near the mitral valve (Fig. 3A and B). The left axillary artery was exposed through a left subclavicular incision, and a median re-sternotomy was performed. Cardiopulmonary bypass was established by anastomosing the graft of the left axillary artery and performing bicaval cannulation. The ascending aorta was clamped, and myocardial protection was achieved using an antegrade cold blood cardioplegic solution. The pseudo-false aneurysm was confirmed by ultrasound to be located in the inferior wall and opened on the right side of the posterior descending artery. The aneurysm comprised an intramyocardial heavy hematoma and necrotic myocardium, and a patch in the RV was observed during the first surgery. After removing the hematoma and debriding the necrotic tissue, the posterior ventricular septal wall defect measured 30 × 50 mm. The defect was closed using a Dacron patch with interrupted mattress sutures around the ridge of the defect, and a second running suture was used to ensure a secure anastomosis (Fig. 3C). The ventricular edge was then closed with interrupted sutures using two Teflon felt strips to externally reinforce the suture. A second running suture was used to ensure a secure right ventriculotomy closure. Finally, cardiopulmonary bypass was weaned.

The patient was extubated the following days, and the IABP was smoothly removed. Postoperative-enhanced CT revealed no pseudo-false aneurysm between the left and right ventricles (Fig. 3D). The patient was discharged without any complications 3 weeks postoperatively.

## Discussion and conclusions

VSP in acute myocardial infarction is classified into the simple type, which is perforated directly from the LV to the RV, and the complex type, which is dissected by a serpiginous path in the septal myocardium and perforated into the RV. The complex type constitutes 21% of anterior wall infarctions and 69% of inferior wall infarctions, which makes it more common in inferior wall infarction [3]. Interventricular septal dissection as a mechanical complication in acute myocardial infarction is rare [4, 5]. With interventricular septal dissection, in the absence of a shunt, a left ventricular pseudo-false aneurysm is formed; however, if it is connected to the left and right ventricles, a VSP develops. The mechanisms of formation of false and pseudo-false aneurysms of the LV following myocardial infarction are similar; both occur because of hematoma dissection into the infarcted area. However, false aneurysms are composed of a densely fused pericardium and epicardium, whereas pseudo-false aneurysms are composed of elements of the ventricular wall [6]. In most reported cases, pseudo-false aneurysms develop in the posterior or inferior wall [7]. Otherwise, rupture to the RV is more common [8].

Treatment of VSP following acute myocardial infarction remains challenging. Endocardial patch repair via a left ventricular incision, described as “infarct exclusion,” is a common surgical treatment for postinfarction VSP [9]. Recently, several authors have reported the efficacy of a sandwich patch VSP repair via an RV incision [10–12]. Our patient had VSP with a complex type of inferior wall infarction. We attempted to close the VSP using the right ventricular approach; however, the initial surgery closed only the exit port, and the entry port of the LV could not be closed. Three months postoperatively, the patient developed a large pseudo-false ventricular aneurysm without a shunt, causing hypofunction of the left heart and heart failure.

When performing surgery for the complex type of VSP, the approach from the RV requires sufficient expansion of the VSP, and ensuring that the VSP is closed is necessary. In the present case, we needed to securely remove the extensive fragile necrotic myocardium because we needed to suture all sutures transmurally from the LV to RV. The margin outside the suture appeared to play a role in dispersing the pressure of both the pinching force that was exerted by the sutures and the LV pressure on the patch over a wider area of the myocardium at the edge of the VSP [13]. Hence, a large patch was required to reduce the residual leak.

Eventually, the patient was treated without making an incision in the LV during either surgeries, which might have suppressed the decline in cardiac function. Thus, the left heart function did not decline, and the patient’s cardiac function was maintained. As VSP is an emergency surgery, it is necessary to examine preoperative cardiac ultrasonography findings, diagnose simple and complex VSP types, and consider surgical procedures.

In conclusion, recognizing and identifying the ventricular septal dissection after myocardial infarction are crucial for providing the best treatment and surgical approaches. When ventricular septal dissection is treated using sandwich patch repair via a right ventricular incision, the entry port in the LV must be securely closed with a large patch using transmural mattress sutures.

## Abbreviations

VSP: Ventricular septal perforation
LV: Left ventricle
RV: Right ventricle
CT: Computed tomography
CK: Creatine kinase
IABP: Intra-aortic balloon pumping
